# Investigation into the effect of divergent feed efficiency phenotype on the bovine rumen microbiota across diet and breed

**DOI:** 10.1038/s41598-020-71458-0

**Published:** 2020-09-18

**Authors:** Emily McGovern, Mark McGee, Colin J. Byrne, David A. Kenny, Alan K. Kelly, Sinéad M. Waters

**Affiliations:** 1 Teagasc, Animal and Bioscience Research Department, Animal & Grassland Research and Innovation Centre, Teagasc, Grange, Dunsany, County Meath, Ireland; 2 UCD, College of Health and Agricultural Sciences, University College Dublin, Belfield, Dublin 4, Ireland; 3 Present Address: Microbiome Research Centre, St George & Sutherland Clinical School, University of New South Wales, Sydney, NSW Australia; 4 Present Address: School of Veterinary and Life Sciences, Murdoch University, South Street, Perth, WA 6150 Australia

**Keywords:** Microbiology, Molecular biology, Microbial ecology

## Abstract

The relationship between rumen microbiota and host feed efficiency phenotype, for genetically divergent beef cattle breeds is unclear. This is further exacerbated when different growth stages, chemically diverse diets and production systems are considered. Residual feed intake (RFI), a measure of feed efficiency, was calculated for individually fed Charolais (CH) and Holstein–Friesian (HF) steers during each of four 70-day (excluding adaptation) successive dietary phases: namely, high-concentrate, grass silage, fresh zero-grazed grass and high-concentrate again. Rumen fluid from the ten highest- (HRFI) and ten lowest-ranking (LRFI) animals for RFI, within breed, during each dietary phase was collected using a trans-oesophageal sampler and subjected to 16S rRNA amplicon sequencing and metabolic profiling. The datasets were analysed to identify microbial and rumen fermentation markers associated with RFI status. Age, dietary phase and breed were included in the statistical model. Within breed, for each dietary phase, mid-test metabolic weight and average daily gain did not differ (*P* > 0.05) between HRFI and LRFI steers; however, for the initial high-concentrate, grass silage, fresh grass herbage and final high-concentrate dietary phases, HRFI HF steers consumed 19, 23, 18 and 27% more (*P* < 0.001) than their LRFI counterparts. Corresponding percentages for CH HRFI compared to CH LRFI steers were 18, 23, 13 and 22%. Ten OTUs were associated with RFI (q < 0.05) independent of the other factors investigated. Of these Methanomassiliicoccaceae, *Mogibacteriaceae and* the genus *p-75-a5* of Erysipelotrichaceae *and* were negatively associated (*q* < 0.05) with RFI. The results gave evidence that microbial species could potentially be an indicator of RFI in ruminants rather than broader microbiome metrics; however, further research is required to elucidate this association.

## Introduction

The agricultural sector contributes to about 10–12% of global anthropogenic emissions with enteric methane accounting for 32–40% of this^1^. As a result, improving the efficiency of cattle production will be key to adhering reduction in individual national greenhouse gas emissions under the United Nations Framework Convention on Climate Change Paris Agreement^2^. Beef production can use up to five times more biomass for producing 1 kg of animal protein than dairy^3^ contributing to both an environmental and economic challenges. Feed provision is classed as the single largest variable cost associated with beef production; reducing feed costs through identification of more feed efficient cattle should increase profitability and environmental and economic sustainability of beef farming enterprises^4^. Improvement of feed efficiency (FE) in cattle is not only critical to improve environmental but also for economic sustainability^5,6^. Residual feed intake (RFI) is an established measure of FE in beef cattle, defined as the difference between an animal’s actual compared to its predicted feed intake, usually based on weight and growth^7^. Animals with a low RFI (LRFI) phenotype, eat less than expected and are deemed to be efficient, typically consuming up to 20% less feed than their inefficient high RFI (HRFI) contemporaries, while supporting the same bodyweight and tissue growth^8–10^.

The rumen of cattle is a complex fermentation vat inhabited by a multitude of microbial species acting synergistically to convert plant materials into nutrients, primarily short chain fatty acids (SCFAs) and microbial protein^11^. This process enables ruminants to convert human indigestible plant polysaccharides into high quality meat and milk products suitable for human consumption. The predominate SCFAs, acetate, propionate and butyrate, provide up to 70% of the host’s energy requirements^12,13^. Determination of the relationship between the rumen microbiome and host FE has the potential to facilitate selection of livestock with enhanced nutrient utilization or enable the manipulation of the rumen microbiome to enhance its energy harvesting capacity^14–16^. Methanogenic archaea utilize by-products of rumen fermentation to produce methane which is an energetically wasteful process for the host animal^17^ and a potent greenhouse gas^18^. Diversion of these by-products away from methanogensis and towards alternative biochemical pathways, more beneficial to the host ruminant has been hypothesized as one mechanism to influence host FE^11,14^. Therefore, enhancing feed efficiency has the dual benefit of reducing both feed costs and anthropogenic methane production^19,20^. Currently, however, there is a lack of understanding of the ruminal metabolic and physiological mechanisms underlying variation for RFI in cattle and how this might be impacted upon by genetic makeup of the animal as well as the chemical composition of the diet fed.

The main impediment to genetic progress and adoption of selection for feed efficiency is the logistics and expense of measuring individual animal intake necessary to accurately identify feed efficient animals^21^. Kelly et al.^8^ and Coyle et al.^22^ have demonstrated that RFI phenotype is moderately repeatable for cattle offered the same diet over different phases of their lifetime; however, repeatability is reduced when contrasting diets are fed successively (i.e. forage versus concentrate-based diets), as per commercial farming practice^23^. These results may have implications for beef genetic evaluations and breeding programmes. Shabat et al.^14^ reported that rumen microbiome genes and species could predict the variation in a dairy cow’s RFI phenotype (91% accuracy). Thus, if a microbial marker for FE could be deduced there is potential to utilize it for genomic selection of feed efficient cattle. However, the study by Shabat et al.^14^ was conducted across a single timepoint, using Holstein Friesian dairy cattle and on a concentrate diet, leaving the resilience of microbial markers across breed, dietary phase and physiological age of the ruminant yet to be investigated.

The current study investigated the effect of RFI phenotype of both beef and dairy bred steers across common dietary phases of Irish pastoral-based beef production systems on the rumen microbiome and its metabolites. This aims to elucidate if particular microbial taxa influence host FE phenotype independent of stage of production, diet and host breed, leading to the potential for rumen microbiome manipulation or selection for FE based on microbial markers.

## Results

### Animal performance

Descriptive statistics of DMI, RFI, ADG and MBW are presented separately for each breed type. For high-concentrate, grass silage, fresh grass herbage and (second) high-concentrate dietary phases, HRFI HF steers consumed 19, 23, 18, and 27% more (*P* < 0.001) than their low RFI counterparts, respectively (Table 1). Corresponding percentages for HRFI compared to LRFI CH steers were 18, 23, 13 and 22%. In each dietary phase within breed, mid-test MBW and ADG did not differ (*P* > 0.05) between the RFI groups (Table 1).

**Table 1:** Dry matter intake (DMI) (kg/day), residual feed intake (RFI), average daily gain (ADG) (kg) and Mid-test metabolic weight (MBW) (kg) for CH and HF steers ranked low (LRFI) and high (HRFI) RFI offered; high-concentrate, grass silage and zero-grazed grass and a second high-concentrate diet. *P* values are derived using a Wilcoxon rank sum test to assess the differences between treatments.

Trait	High concentrate 1						Grass silage					Zero grazed grass					High concentrate 2				
	Breed	LRFI	S.D	HRFI	S.D	*P* value	LRFI	S.D	HRFI	S.D	*P* value	LRFI	S.D	HRFI	S.D	*P* value	LRFI	S.D	HRFI	S.D	*P* value
DMI	CH	7.71	0.66	9.06	0.20	< 0.000	5.87	0.49	6.99	0.41	< 0.001	8.56	0.50	9.69	1.65	< 0.001	10.49	0.75	12.85	1.00	< 0.001
	HF	7.99	0.89	9.51	0.26	< 0.001	6.31	0.40	7.77	0.24	< 0.001	8.51	0.47	10.07	0.23	< 0.001	11.18	1.29	14.28	1.09	< 0.001
RFI	CH	− 0.81	0.13	0.82	0.19	< 0.001	− 0.60	0.16	0.59	0.12	< 0.001	− 0.56	0.14	0.58	0.67	< 0.001	− 1.14	0.26	1.01	0.19	< 0.001
	HF	− 0.91	0.29	0.69	0.19	< 0.001	− 0.80	0.23	0.82	0.32	< 0.001	− 0.82	0.24	0.73	0.13	< 0.001	− 1.81	0.49	1.37	0.28	< 0.001
ADG	CH	1.42	0.28	1.33	0.06	NS	0.38	0.23	0.34	0.13	NS	1.35	0.13	1.37	0.37	NS	1.38	0.26	1.52	0.27	NS
	HF	1.42	0.23	1.42	0.04	NS	0.52	0.18	0.55	0.18	NS	1.24	0.34	1.17	0.23	NS	1.36	0.52	1.29	0.18	NS
MBW	CH	432.09	34.23	426.81	25.40	NS	488.62	26.61	494.25	30.15	NS	565.48	46.88	563.16	45.26	NS	733.37	51.90	736.70	58.24	NS
	HF	348.45	47.69	348.74	43.25	NS	415.05	32.56	398.94	24.78	NS	469.75	18.66	477.07	53.95	NS	683.33	86.47	680.25	57.99	NS

### Sequence analysis

Amplicon sequencing generated 84,462,720 total reads giving an average of 272,460 ± 69,596 reads per sample. This reduced to 217,817 ± 55,519 when sequences were merged and quality filtered. The average number of counts per sample that were assigned to an OTU (post filtering) was 175,304 ± 74,272. Positive control samples were subjected to the same bioinformatics pipeline as rumen 16S rRNA libraries; generating an average of 203,612 ± 19,378 sequence reads, 141,873 ± 15,724 merged sequences and 132,567 ± 14,397 final counts per sample. The positive controls were positively correlated with the theoretical composition of the microbial community standard DNA (*P* < 0.05) deeming the laboratory and informatic methodology satisfactory. Negative controls generated 215.3 ± 107 final counts per sample.

### Rumen fermentation profile

Rumen SCFA analysis showed that LRFI CH steers had lower concentrations of propionate and total SCFA in their rumen fluid in comparison to HRFI CH steers (*P* < 0.05) when offered a high-concentrate diet during their growth phase (Table 2). In HF steers offered the same diet, LRFI steers had a higher concentration of acetate, increased total SCFA concentration in comparison to HRFI steers (Table 2). No differences in any metabolite concentrations existed between HRFI and LRFI steers offered a grass silage diet for either the CH or HF breed (*P* > 0.05) (Table 2). LRFI CH steers had reduced butyrate concentration (*P* < 0.01) HRFI CH steers offered a zero-grazed grass diet, while there was an increase in acetate, propionate, isobutyrate, valerate and total SCFA concentrations in LRFI steers in comparison to HRFI HF steers offered a zero-grazed grass diet (*P* < 0.05) (Table 2). There was an increase in isovalerate concentrations in LRFI CH steers in comparison to HRFI CH (*P* < 0.1) and no differences in SCFA concentrations existed between LRFI and HRFI HF steers fed a high-concentrate diet during the finishing phase (*P* > 0.05, Table 2).

**Table 2:** Short chain fatty acid concentrations (mmol/L) (A) CH and (B) HF steers ranked low (LRFI) and high (HRFI) RFI offered common dietary phases of Irish pastoral-based beef production systems; high-concentrate, grass silage and zero-grazed grass and a second high-concentrate diet during finishing phase. *P* values are derived using a Wilcoxon rank sum test to assess the differences between treatments.

Dietary Phase	High concentrate 1					Grass silage					Zero grazed grass					High concentrate 2				
	HRFI	S.D	LRFI	S.D	P value	HRFI	S.D	LRFI	S.D	*P* value	HRFI	S.D	LRFI	S.D	*P* value	HRFI	S.D	LRFI	S.D	*P* value
(A)																				
Acetic	65.8	7.5	59.8	5.5	NS	57.9	7.3	62.7	14.9	NS	58.0	6.6	58.1	6.1	NS	63.6	11.2	66.9	9.2	NS
Propionic	33.4	4.8	27.0	5.3	< 0.05	13.0	3.4	15.0	4.6	NS	10.8	1.7	9.8	1.3	NS	22.2	4.8	24.1	5.6	NS
Isobutyric	0.8	0.2	1.0	0.4	NS	1.2	0.3	1.4	0.3	NS	1.8	0.3	1.8	0.2	NS	0.9	0.4	1.1	0.5	NS
Butyric	7.9	1.4	7.5	1.7	NS	10.9	3.0	11.4	2.8	NS	9.8	2.3	7.9	1.3	< 0.01	12.1	4.2	12.7	3.0	NS
Isovaleric	0.9	0.3	1.3	0.6	NS	1.9	0.5	1.7	0.2	NS	1.8	0.5	1.8	0.3	NS	1.5	0.7	2.6	1.4	< 0.1
Valeric	2.6	0.6	2.3	0.5	NS	1.7	0.4	1.4	0.1	NS	1.1	0.2	0.9	0.1	NS	2.9	1.0	2.7	0.6	NS
A:P ratio	2.0	0.2	2.3	0.6	NS	4.7	1.1	4.3	0.4	NS	5.4	0.5	5.9	0.6	NS	3.0	0.8	3.0	1.1	NS
Total SCFA	111.4	12.1	98.8	7.6	< 0.05	86.5	11.9	93.6	22.3	NS	83.3	10.0	80.4	8.1	NS	103.1	15.1	110.1	10.2	NS
(B)																				
Acetic	59.0	5.6	67.9	8.3	< 0.05	54.5	5.3	58.0	5.1	NS	53.7	4.5	63.1	10.2	< 0.05	65.2	6.7	72.0	11.8	NS
Propionic	33.6	7.2	36.9	5.3	NS	13.6	1.0	14.1	2.4	NS	9.7	0.7	12.1	2.2	< 0.05	20.8	5.1	25.1	6.9	NS
Isobutyric	0.8	0.2	0.9	0.3	NS	1.2	0.1	1.3	0.2	NS	1.6	0.2	2.0	0.2	< 0.05	1.2	0.4	1.0	0.4	NS
butyric	9.2	6.3	9.2	2.1	NS	9.1	1.3	10.1	2.8	NS	8.5	1.4	10.1	2.4	< 0.1	12.9	3.7	13.1	5.1	NS
Iso valeric	0.9	0.4	1.1	0.7	NS	1.5	0.3	1.6	0.4	NS	1.7	0.3	1.9	0.3	NS	2.4	0.9	1.9	0.4	NS
Valeric	2.5	0.6	3.1	0.6	NS	1.5	0.3	1.5	0.3	NS	1.0	0.1	1.3	0.2	< 0.01	2.4	0.6	2.9	1.1	NS
A:P ratio	1.9	0.6	1.9	0.3	NS	4.0	0.3	4.2	0.5	NS	5.6	0.6	5.2	0.5	< 0.05	3.3	0.7	3.1	1.1	NS
Total SCFA	105.9	11.2	119.1	12.9	< 0.05	81.4	6.4	86.6	9.5	NS	76.1	5.9	90.4	14.4	< 0.05	104.9	10.4	116.0	16.4	NS

Overall no consistent rumen fermentation profile was observed between divergent RFI phenotypes across the phases investigated in the study (Table 2). The association between SCFA concentration and RFI was investigated, highlighting dietary phase, age and breed as potential confounding factors. No association between RFI and rumen fermentation profile was observed (q > 0.05).

### Ordination analysis of microbial community structure

**Figure 1:**
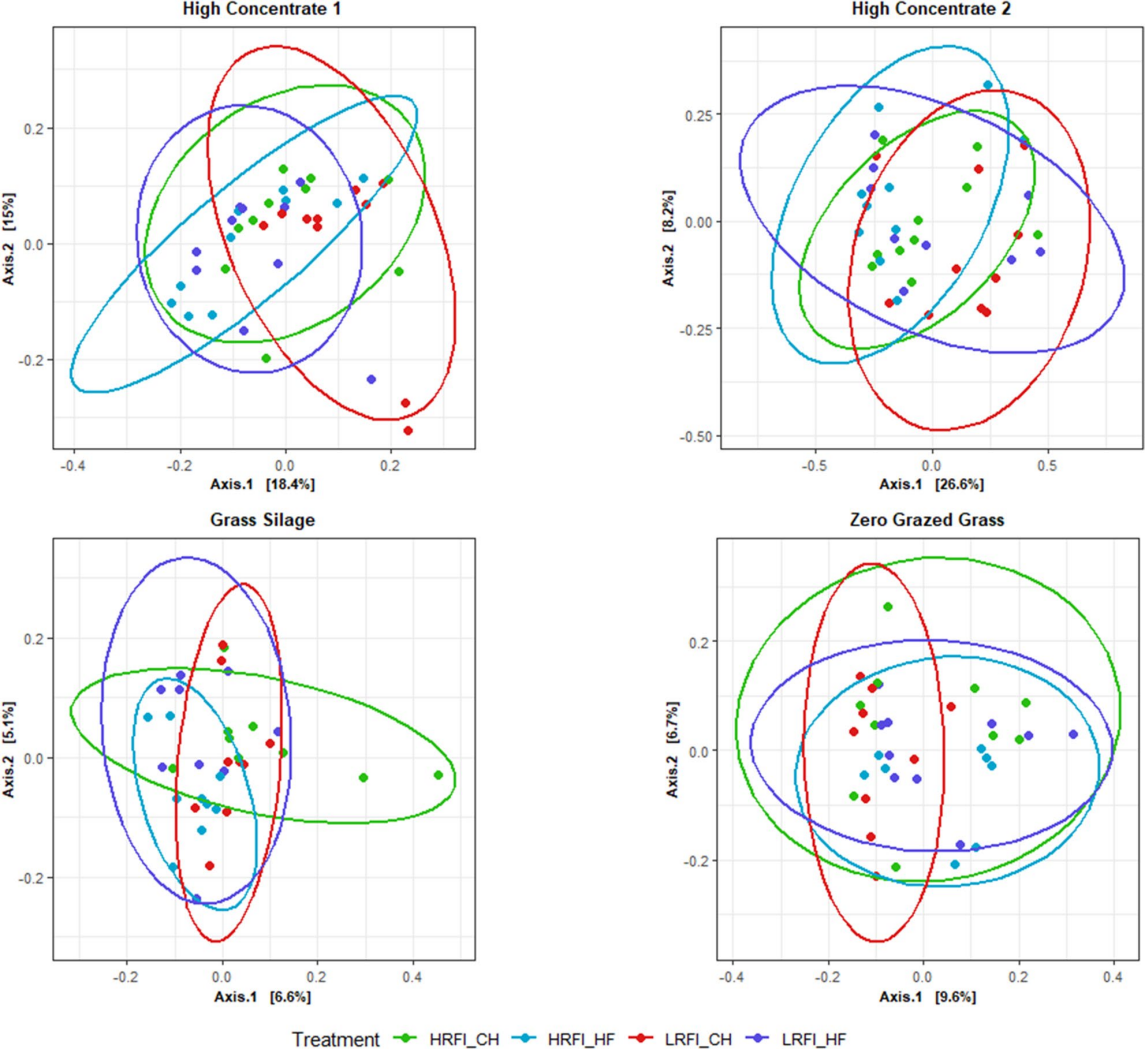
Principal component ordination analysis (PCoA) plot indicating similarity bacterial and archaeal community of Charolais (CH) and Holstein Friesian (HF) divergent for residual feed intake (RFI) steers offered; high concentrate (C1), grass silage (GS) and zero grazed grass (ZGG) and a second high-concentrate diet (C2). This is based on similarity of OTU composition of each sample calculated using Bray–Curtis similarity index and plotted using Principal component ordination analysis.

**Figure 2:**
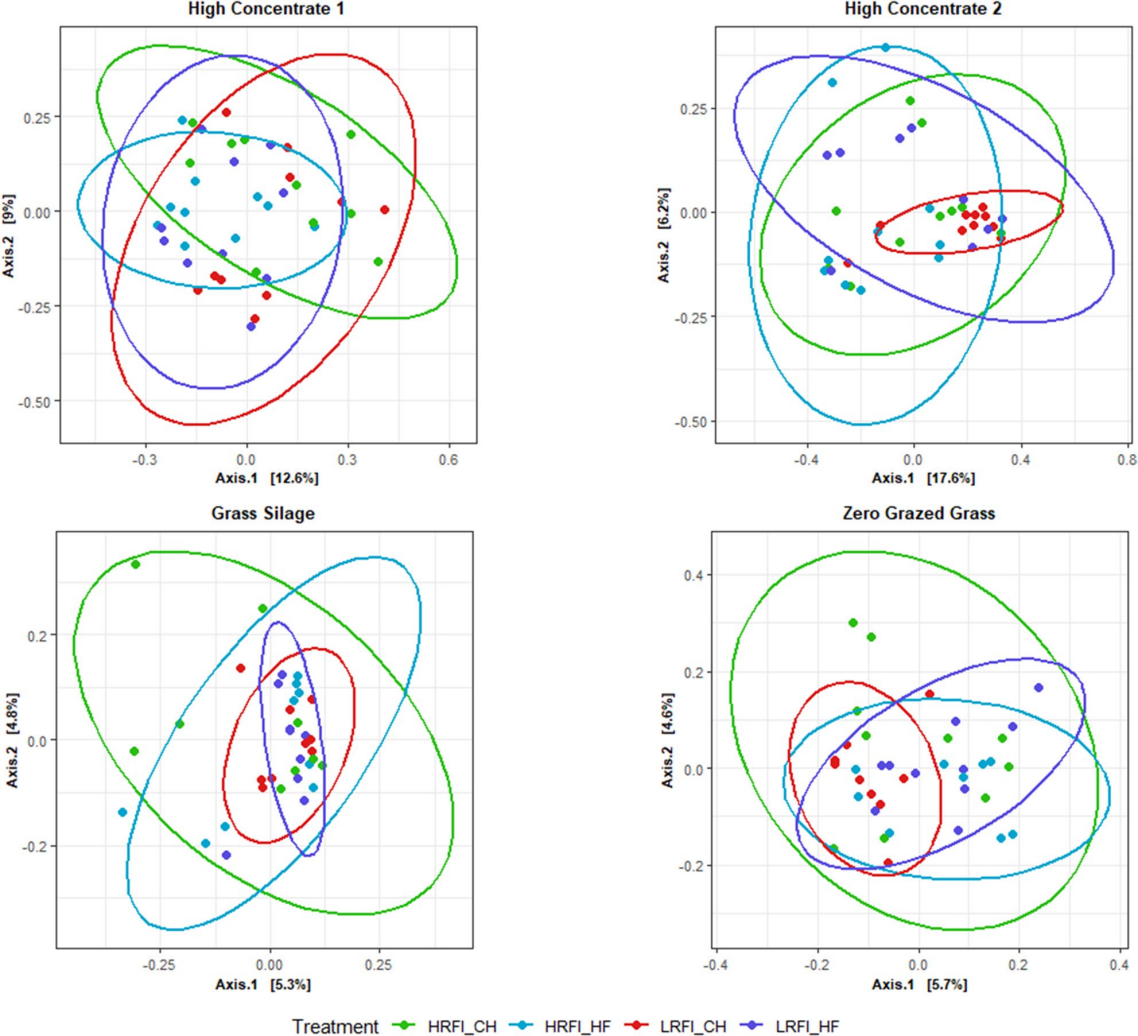
Principal component ordination analysis (PCoA) plot indicating similarity bacterial and archaeal community of Charolais (CH) and Holstein Friesian (HF) divergent for residual feed intake (RFI) steers offered; high concentrate (C1), grass silage (GS) and zero grazed grass (ZGG) and a second high concentrate diet (C2). This is based on similarity of OTU composition of each sample calculated using Weighted Unifrac distance metric and plotted using principal component ordination analysis.

Principal component ordination analysis (PCoA) analyses (Fig. 1) were used to visualise the similarities and diversity of bacterial and archaeal populations of HRFI and LRFI steers across dietary phase and breed. The plot showed a lack of clustering based on RFI phenotype. Samples clustered tightly based on diet offered and dietary phase. Pairwise comparisons using Bray Curtis and Unifrac dissimilarity metrics between RFI phenotype within each diet and breed and comparisons between breed with the same RFI phenotype within dietary phase are summarised in Table 3 and visualised in Figs. 1 and 2. In summary, bacterial community structure did not differ between RFI phenotype in steers in any experimental phase irrespective of breed (*P* > 0.05) (Table 3).

**Table 3:** Effect of residual feed intake (RFI) phenotype and breed on beta diversity for Charolais (CH) and Holstein Friesian (HF) steers ranked low (LRFI) and high (HRFI) RFI offered common dietary phases of Irish pastoral-based beef production systems; high-concentrate, grass silage and zero-grazed grass during growth phases and a second high-concentrate diet. Distance based permutation multivariate analysis of variance (PERMANOVA) was performed to test the null hypothesis that there were no differences in the microbial community structure across treatment at a significance level of *P* = 0.05 based on 999 permutations.

Factor	Group 1	Group 2	*n*	Weighted Unifrac *P* value	Weight Unifrac q value	Bray *P* value	Bray q value
RFI	HRFI_C1_CH	LRFI_C1_CH	20	0.30	0.32	0.25	0.27
RFI	HRFI_C1_HF	LRFI_C1_HF	20	0.49	0.50	0.90	0.90
RFI	HRFI_C2_CH	LRFI_C2_CH	20	0.10	0.11	0.09	0.10
RFI	HRFI_C2_HF	LRFI_C2_HF	19	0.33	0.34	0.29	0.31
RFI	HRFI_GS_CH	LRFI_GS_CH	18	0.09	0.10	0.25	0.27
RFI	HRFI_GS_HF	LRFI_GS_HF	18	0.42	0.43	0.19	0.21
RFI	HRFI_ZGG_CH	LRFI_ZGG_CH	19	0.06	0.07	0.10	0.11
RFI	HRFI_ZGG_HF	LRFI_ZGG_HF	19	0.78	0.78	0.49	0.49
Breed	HRFI_C1_CH	HRFI_C1_HF	20	0.04	0.05	0.14	0.15
Breed	HRFI_C2_CH	HRFI_C2_HF	20	0.13	0.14	0.22	0.24
Breed	HRFI_GS_CH	HRFI_GS_HF	18	0.00	0.00	0.00	0.00
Breed	HRFI_ZGG_CH	HRFI_ZGG_HF	19	0.11	0.12	0.10	0.11
Breed	LRFI_C1_CH	LRFI_C1_HF	20	0.04	0.04	0.02	0.02
Breed	LRFI_C2_CH	LRFI_C2_HF	19	0.24	0.25	0.45	0.45
Breed	LRFI_GS_CH	LRFI_GS_HF	18	0.28	0.30	0.11	0.12
Breed	LRFI_ZGG_CH	LRFI_ZGG_HF	19	0.01	0.01	0.02	0.02

### Alpha diversity

Similar to the beta diversity results, there was no difference in alpha diversity observed in the rumen microbiome of steers with divergent RFI phenotypes in any phase or for either breed (Table 4). Maaslin2 was also used to investigate if alpha diversity index showed an association with RFI phenotype. However, no alpha diversity metrics were significantly associated with RFI regardless of phase, breed or age (*q* < 0.05).

**Table 4:** Effect of high RFI (HRFI) and low RFI (LRFI) phenotype on species presence, phylogenetic diversity (Faith_PD), species richness, species evenness and Shannon diversity for Charolais (CH) and Holstein Friesian (HF) steers offered high-concentrate, grass silage and zero-grazed grass during growth phases and a second high-concentrate diet. *P* values are derived using a Wilcoxon rank sum test to assess the differences between treatments.

	Breed	High-concentrate 1			Grass silage			Zero-grazed grass			High-concentrate 2		
		HRFI	LRFI	*q* value	HRFI	LRFI	*q* value	HRFI	LRFI	*q* value	HRFI	LRFI	*q* value
Species	CH	615	652	NS	2121	2266	NS	2141	2375	NS	735	848	NS
	HF	516	581	NS	2328	2447	NS	2269	2073	NS	566	723	NS
Phylogenetic Diversity	CH	54.24	50.97	NS	85.95	82.59	NS	81.49	86.75	NS	45.06	50.80	NS
	HF	44.31	48.38	NS	91.84	88.27	NS	82.66	78.81	NS	41.10	47.61	NS
Evenness	CH	0.58	0.59	NS	0.81	0.82	NS	0.80	0.82	NS	0.67	0.68	NS
	HF	0.59	0.61	NS	0.82	0.82	NS	0.81	0.80	NS	0.64	0.67	NS
Shannon	CH	5.38	5.48	NS	8.92	9.15	NS	8.87	9.20	NS	6.41	6.65	NS
	HF	5.29	5.58	NS	9.21	9.23	NS	9.05	8.83	NS	5.79	6.36	NS

### Effect of RFI phenotype on the archaeal and bacterial populations

No differentially abundant microbial taxa were identified between RFI phenotypes. In total there were 25 OTUs associated with RFI (*q* < 0.05) with 10 of these associated with RFI independent of any other factor investigated (i.e. Phase, Breed, Age). These are summarised in Table 5.

**Table 5:** Correlation between identified taxa and residual feed intake (RFI) using multivariate Association with Linear Models analysis.

Level	Classification	value	coef	Std err	N	N_not_0	*P* value	*q* value
OTU	*Oribacterium*	RFI	0.0012	0.0004	152	105	0.005	0.076
OTU	*P-75-a5*	RFI	− 0.0007	0.0002	152	135	0.004	0.058
OTU	*WCHB1-25*	RFI	− 0.0010	0.0002	152	90	0.000	0.003
OTU	*Methanomassiliicoccaceae*	RFI	− 0.0010	0.0003	152	133	0.001	0.030
OTU	*GMD14H09*	RFI	− 0.0012	0.0004	152	122	0.006	0.089
OTU	*Mogibacteriaceae*	RFI	− 0.0022	0.0007	152	152	0.001	0.023
OTU	*S24-7*	RFI	− 0.0043	0.0013	152	152	0.001	0.031
OTU	*Ruminobacter*	RFI	− 0.0051	0.0017	152	52	0.003	0.055
OTU	*Weissella*	RFI	− 0.0002	0.0001	152	9	0.002	0.045
OTU	*Lactobacillales*	RFI	− 0.0003	0.0001	152	3	0.003	0.049
Genus	*S24-7*	RFI	− 0.0067	0.0020	152	152	0.001	0.020
Genus	*Mogibacteriaceae*	RFI	− 0.0035	0.0010	152	152	0.001	0.020
Genus	*Methanomassiliicoccaceae*	RFI	− 0.0016	0.0005	152	133	0.001	0.022
Genus	*Ruminobacter*	RFI	− 0.0078	0.0025	152	52	0.002	0.037
Genus	*p-75-a5*	RFI	− 0.0011	0.0004	152	135	0.003	0.042
Genus	*Lactobacillales*	RFI	− 0.0004	0.0001	152	3	0.003	0.047
Genus	*GMD14H09*	RFI	− 0.0018	0.0007	152	122	0.006	0.081

## Discussion

Feed efficiency is an environmentally and economically important driver sustainable beef production^24^. The rumen microbial community mediates energy available to the host ruminant through its fermentative activity, which suggests it plays a fundamental role in feed efficiency^21^. Previous studies have identified that host FE phenotype influences the rumen microbiome and rumen fermentation^14,25,26^. However, a key challenge is to identify whether these relationships are consistent across animal breed, physiological age and dietary phase. This study therefore examined the effect of RFI phenotype on ruminal fermentation profile and bacteria and archaeal populations in CH and HF steers, across four dietary phases.

Ruminal SCFA parameters are indicative of active bacterial fermentation and host rumen epithelial absorption^27^. It is presumed that due to the central role of ruminal digestion in the supply of nutrients for post absorptive metabolic processes, that differences in rumen fermentation profile would be observed between HRFI and LRFI phenotypes^14,28^. Despite this, there was no consistent relationship observed across breed and dietary phase between individual rumen metabolites and RFI rank for steers. Furthermore, fermentation profiles of the steers of the same breed and the same feed efficiency phenotype, offered the same diet during different production phases (i.e. high-concentrate 1 and high-concentrate 2) also showed no consistency in fermentation profile. This result is in agreement with previous studies which highlight the lack of consistency of the rumen fermentation profiles of cattle divergent for feed efficiency, as reviewed by Kenny, et al.^21^. Results do however, indicate that dietary phase has a more pronounced influence on bacterial fermentation profiles than RFI phenotype, with SCFA profiles observed consistent with expected proportions based on dietary composition, supporting previous findings by our group^29^ and others^30^.

Shabat et al.^14^ hypothesised that a LRFI phenotype supports an efficient rumen microbiome which has lower diversity and richness than its HRFI counterparts, which is also confirmed by the work of Guan et al.^31^. The efficient rumen microbiome is less diverse and produces a smaller range of “relevant” output metabolites suitable for energy and carbon channelling to the animal i.e. meeting the ruminant’s energetic needs, while lowering methane emissions to the atmosphere^14^. There is some ambiguity, however, in the literature regarding this hypothesis, as it is contradicted by the studies of Paz et al.^25^, Li^16^, Myer et al.^32^ and indeed our own previous findings^33^ which reported no differences in microbial diversity between HRFI and LRFI animals. In agreement with much of the literature, no decrease in diversity or richness was observed for any steers deemed as feed efficient relative to their inefficient contemporaries of the same breed offered the same diet in the current study. These results indicate that a lower diversity microbiome may not necessarily equate to a more feed efficient ruminant and that microbial diversity is influenced by the chemical composition of the prevailing diet and breed as previously reported in the literature^30^.

Although there have been some reports of differences in overall community structure in relation to RFI^14,31^, research from this study indicates that it is more likely that individual microbial species or strains may impact the efficiency of the animal more than an overall ruminal community shift. The association analysis provided some evidence of the influence of RFI phenotype on rumen microbial populations, across all dietary phases and breed. Rumen methanogen populations have been associated with RFI in ruminants^19,34,35^, with efficient livestock identified to produce lower methane yields compared to their inefficient contemporaries^19,20^. Methanogen populations in the rumen are responsible for methane production; an energetically wasteful process for the host ruminant, therefore it has been hypothesized that increased abundance of methanogenic archaea reduce the feed efficiency status of the host ruminant^36^. In contrast, in the current study a negative relationship was observed between an archaeal members of the Methanomassiliicoccaceae family exhibited a negative relationship with the RFI trait at genus and OTU level. The Methanomassiliicoccaceae family are obligate H_2_-dependent methylotrophs, utilising methyl groups from methanol and methylamines (mono-, di-, and tri-methylamine) and methyl thiols for the production of methyl coenzyme^37^ and provide additional NH_4_^+^ to rumen bacteria. Li^16^ found that Methanomassiliicoccales tended to be more abundant in low compared with high RFI cattle. In different experimental ruminant models, McGovern et al.^38^ and Danielsson et al.^39^ both observed a decrease in Methanomassiliicoccaceae when members of the *Methanobrevibacter* gottschalkii/SGMT clade increased and *Methanobrevibacter* ruminantium/RO clade decreased. *Methanobrevibacter* is the most common hydrogenotrophic archaeal genus and can be divided into two subgroups, one known as the gottschalkii /SGMT clade (*Mbb*. smithii, *Mbb.* gottschalkii, *Mbb.* Millerae and *Mbb.* thaueri), the other ruminantium/RO clade comprising principally of *Mbb.* Ruminantium and *Mbb.* Olleyae^40^. Kittelmann et al.^41^ previously compared the relative abundances of the two clades and showed they have a negative relationship (*R*^2^ = 0.51). These methanogen clades have different affinities for hydrogen (H_2_), with the SGMT clade possessing methyl coenzyme M reductase (MCR) isozymes; MCR I and MCR II^42^ which enables members of the SGMT clade to utilise H_2_ at higher concentrations, compared to the RO clade that possess only MCR I^42,43^. Methanomassiliicoccaceae*,* similar to the RO clade, thrive in a lower H_2_ environment. Therefore, it is more likely that the composition of the archaeal community rather than total methanogen abundance may have greater significance with respect to feed efficiency^38^. It has also been hypothesized that the Methanomassiliicoccales order may have a protective effect in the rumen of LRFI steers, eliminating the potential negative effects of high methylamine concentrations^44,45^ and also providing additional NH_4_^+^ for nitrogen cycling^46^*.*

The association between bacterial genera and RFI less clear with very few of the bacterial species shown to be associated with the trait. A Mogibacteriaceae-affiliated unnamed OTU which was found to be negatively correlated with RFI, *Mogibacterium* are asaccharolytic, Gram positive bacteria^47^ and are hypothesised to be involved with ammonia assimilation^48^. *Mogibacterium* utilises ammonia and produces phenylacetate, a precursor for phenylalanine^47^. Previously a Mogibacteriaceae-affiliated genus was reported to be associated with feed efficiency in beef cattle with multiple genetic breeds^32^. Despite the lack of information pertaining to this bacterial genera and its contribution to increased FE, there is evidence of increased nitrogen digestion in LRFI cattle^19,49^. Abundance of members in this family were negatively correlated with body mass index (BMI) in humans^50,51^. Cattle with LRFI phenotype have been observed to have leaner carcasses^52^, suggesting the higher abundance of Mogibacteriaceae in L-RFI individuals may correspond to a lean phenotype. It was also observed that an OTU identified as Erysipelotrichaceae *p-75-a5* maintained a negative association with RFI across breed and phase. In a previous study by Li et al.^53^ also investigating the impact of breed on the microbiome and its association with feed efficiency, it was reported that abundance of *p-75-a5 was* elevated in LRFI Charolais steers in comparison to their HRFI contemporaries, however, this elevation was not observed in the other breeds investigated.

## Conclusion

Research to date suggests there is potential to enhance nutrient utilization from feed and improve FE by altering the rumen microbiome. However, there is a lack of information surrounding the effects of RFI phenotype on the rumen microbiome, particularly during varying stages of production when cattle, are offered contrasting diet types. The results of rumen metabolite profiling were inconsistent and showed little to no consistency with RFI phenotype. This study provides evidence that the composition of the methanogenic community present in the rumen may prove to be an indicator of host RFI; however, further comprehensive investigation focusing on archaeal communities at multiple time points will be required to fully elucidate relationship. These results emphasise that targeted metabolite profiling or molecular surveying of the phylogenetic diversity within the rumen may not comprehensively identify microbial or fermentation factors which influence RFI phenotype. This indicates that global high depth metagenomic shotgun sequencing approach is most likely required to elucidate the intricacies of both the diversity and functionality of the rumen microbiome in relation to FE.

## Materials and methods

All procedures involving animals in this study were approved by the Teagasc Animal Ethics Committee and conducted under an experimental license approved by the Irish Health Products Regulatory Authority, project authorisation number AE19132/P029. All methods and experimental protocols in this study were performed in accordance with relevant guidelines and regulations.

### Animal model

This experiment was conducted as part of a larger study designed to examine the within-animal repeatability of intake, growth and FE between the growing and finishing stages of the lifespan of Charolais (CH) and Holstein-Friesian (HF) beef steers offered either the same diet or diets contrasting in energy density and chemical composition^10,22,23^.

**Figure 3:**
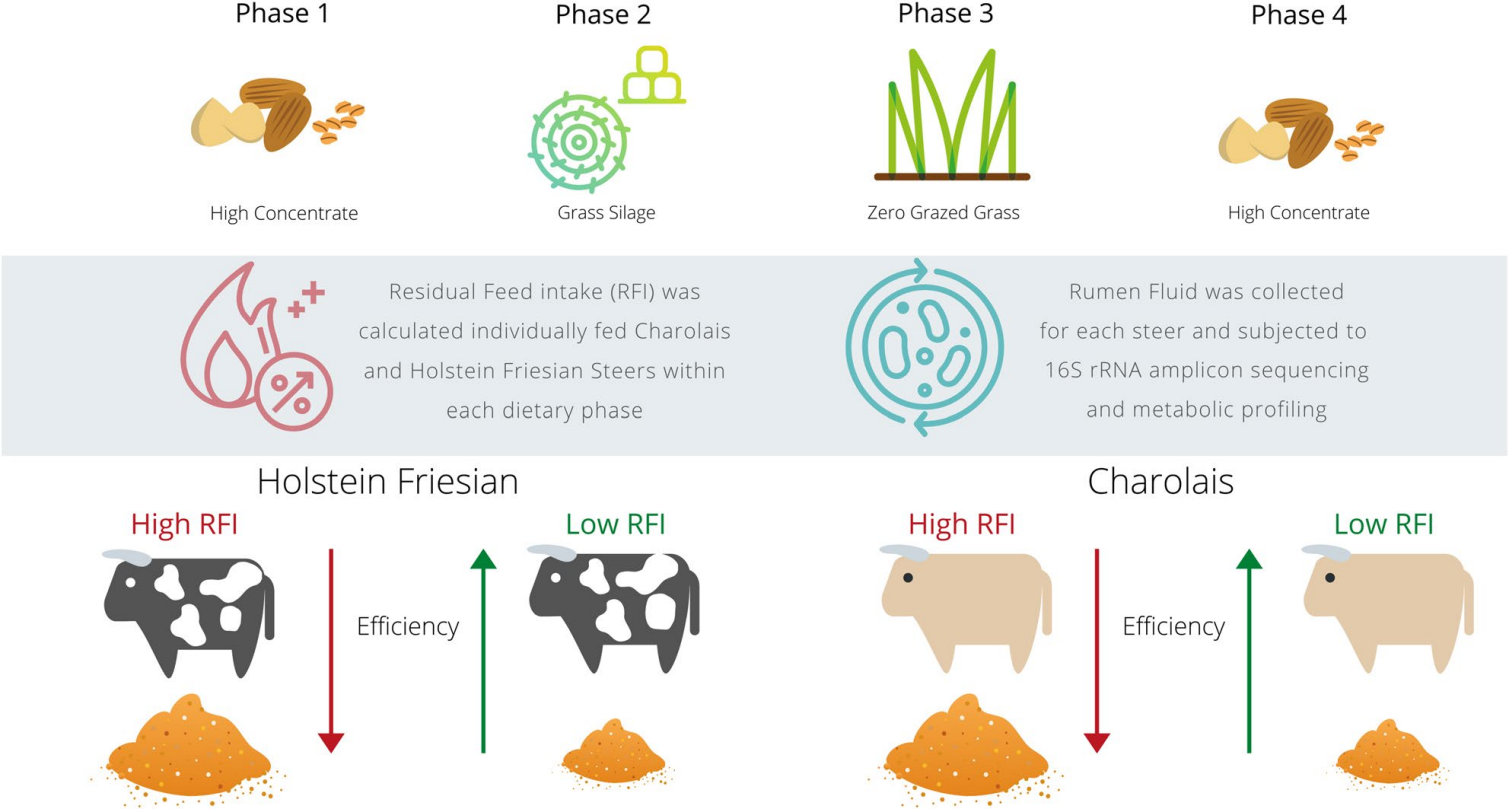
Residual feed intake (RFI) was calculated for Charolais (n = 90) and Holstein–Friesian (n = 77) steers during each of four separate 70 day dietary phases; high-concentrate diet, grass silage, fresh grass herbage and a high-concentrate diet. Rumen fluid samples collected via trans-oesophageal sampler from the 10 highest- and 10 lowest-ranking animals for RFI, within breed, during each dietary phase were used for subsequent metabolomic and 16S rRNA amplicon analysis. Created with Canva **(**https://www.canva.com/).

Briefly, 167 cattle comprising of 90 CH and 77 HF were used in this study. Following a dietary adaptation period, individual dry matter intake (DMI) and growth were measured over four 70-day feeding phases (Fig. 3). Mean BW (SD) and age (SD) at the start of feeding phase 1 (high-concentrate 1) were 394 kg (37.5) and 283 days (18.3), and 294 kg (41.8) and 307 days (7.7), for CH and HF, respectively. Corresponding BW at the start of phase 2 (grass silage) were 485 kg (37.6) and 519 kg (38.3), phase 3 (zero-grazed fresh grass) were 516 kg (37.3) and 440 kg (41.8) and phase 4 (high-concentrate 2) were 676 kg (50.0) and 611 kg (49.1). During the two high-concentrate feeding phases 1 (‘yearling’) and 4 (‘two-year old’) steers were individually offered the same concentrate diet (860 g/kg rolled barley, 60 g/kg soya bean meal, 60 g/kg molasses, and 20 g/kg minerals and vitamins) ad libitum plus a restricted allowance of grass silage daily. Phase 2 consisted of offering grass silage (precision-chop harvested from a primary growth sward, which comprised mainly of perennial ryegrass) to appetite. During phase 3 steers were individually offered zero-grazed grass (DM 196 g/kg) ad libitum. The grass herbage was harvested (without chopping) twice daily from *Lolium perenne* dominant swards using a ‘zero-grazer’ (Model AB70 Zero Grazer, Dromone, Oldcastle, Co. Meath). At the midpoint of each experimental phase after a dietary adaptation perios a single rumen fluid sample was collected from each animal via stomach intubation (Flora Rumen Scoop, Profs-Products, Guelph, Canada) approximately 2 to 4 h post-feeding^20^, for metabolite profiling and microbial analysis. Rumen fluid samples were immediately snap frozen in liquid nitrogen and subsequently stored at – 80 °C for molecular analyses.

### RFI calculation

At the end of each dietary phase, average daily live weight gain (ADG) of each steer was computed as the coefficient of the linear regression of BW (kg) on time (days) using the GLM procedure of SAS 9.3 (SAS Inst. INC., Cary, NC). Mid-test metabolic weight (MBW) was determined as BW^0.75^ 35.5 days prior to the end of the test, which was estimated from the intercept and slope of the regression line after fitting a linear regression through all BW^0.75^ observations. RFI was calculated for each animal as the difference between actual DMI and expected DMI. Expected DMI was computed within breed for each animal using a multiple regression model, regressing DMI on MBW and ADG. Animals were ranked according to RFI coefficient and the 10 highest HRFI and 10 lowest LRFI ranking animals for RFI, within breed, during each dietary phase were used for this study.

For logistical reasons seven samples could not be obtained during the sampling process; leaving a sample size of *n* = 153 Phase one: 10 HRFI CH, 10 LRFI CH, 10 HRFI HF, 10 LRFI HF Phase two: 9 HRFI CH, 9 LRFI CH, 9 HRFI HF, 9 LRFI HF Phase three: 10 HRFI CH, 9 LRFI CH, 9 HRFI HF, 10 LRFI HF Phase four: 10 HRFI CH, 10 LRFI CH, 10 HRFI HF, 9 LRFI HF.

### Rumen fermentation profiling

Short chain fatty acid concentrations in rumen fluid samples were measured using a gas chromatograph (model 3800 Varian gas chromatograph). No results were obtained for eight rumen fluid samples leaving a total of *n* = 145 (Phase one: 10 HRFI CH, 10 LRFI CH, 10 HRFI HF, 9 LRFI HF Phase two: 8 HRFI CH, 8 LRFI CH, 8 HRFI HF, 9 LRFI HF Phase three: 10 HRFI CH, 9 LRFI CH, 8 HRFI HF, 8 LRFI HF Phase four: 10 HRFI CH, 10 LRFI CH, 10 HRFI HF, 8 LRFI HF).

### DNA extraction

Approximately 20 g of frozen rumen liquid sample from each animal was considered as representative. Each sample was homogenised to a fine frozen powder under liquid nitrogen using a pestle and mortar and stored at − 80 °C. Approximately 250 mg of homogenized frozen powder was used for DNA extraction. DNA was extracted using the repeated bead beating and column purification method^54^. DNA extractions were also performed on molecular grade water as a negative control. DNA quality was assessed on an agarose gel. DNA extract yield and purity were assessed with two consecutive readings on the Nanodrop 1000 spectrophotometer.

### 16S rRNA amplicon library preparation

Amplicon libraries (*n* = 155) targeting the V4 region of the 16S rRNA gene in bacteria and archaea were generated by PCR amplification. PCR reactions were performed for amplicon libraries with 20 ng of rumen microbial DNA and 515F forward and 806R reverse primers^55^ designed with Nextera overhang adapters, using 1X KAPA HiFi HotStart ReadyMix DNA polymerase (Roche Diagnositics, West Sussex, UK). Libraries were also generated for a positive control; ZymoBIOMICS Microbial Community Standard DNA (Zymo Research Corp, Irvine, CA, USA) and negative extraction (molecular grade water) controls respectively. Cycle conditions were 95 °C for 3 min, 20 PCR cycles; 95 °C for 30 s, 55 °C for 30 s, 72 °C for 30 s and then 72 °C for 5 min. Amplicons were purified using Qiaquick PCR Purification Kit (Qiagen, Manchester, UK). A second PCR step attached dual indices and Illumina sequencing adapters using Nextera XT index kit. Cycle conditions were 95 °C for 3 min, 8 PCR cycles; 95 °C for 30 s, 55 °C for 30 s, 72 °C for 30 s and then 72 °C for 5 min. Amplicon generation was validated through visualisation on a 2% (w/v) agarose gel. Amplicons were pooled in equal concentrations and gel purified to remove unwanted products using the Qiagen Gel Extraction Kit (Qiagen, Manchester, UK). An extra purification step using the QIAquick purification kit (Qiagen, Manchester, UK) was performed to remove residual agarose. The pooled purified libraries were measured for purity and quantity on the Nanodrop 1000 and further quantified using the KAPA SYBR FAST universal kit with Illumina Primer Premix (Roche Diagnositics, West Sussex, UK). The library pool was then diluted and denatured as recommended by Illumina MiSeq library preparation guide. The sequencing was conducted using 500 cycle MiSeq reagent kits (Illumina, San Diego, CA, USA).

### Sequence analysis

Two samples (HRFI HF, LRFI CH) from phase 3 were removed from the analysis due to low sequence output. The 16S rRNA gene forward and reverse reads were imported into Qiime2^56^. The DADA2 pipeline^57^ was used for detecting and correcting Illumina amplicon sequences, removal of primers and chimeric reads, and assembly into sequence variants (SV)/operational taxonomic units (OTUs)^58^. Taxonomy was assigned using a naïve Bayes classifier trained on the RefSeq database^59^. Sequence files associated with each sample have been submitted to the NCBI Sequence Read Archive (Accession no. PRJNA483745).

Rumen samples were rarefied to a sampling depth of 49,075 sequences. Alpha-diversity metrics investigated included Faith’s Phylogenetic Diversity (PD), Evenness, Observed Operational Taxonomy Units (OTUs) and Shannon’s diversity index which were calculated using qiime2-q2-diversity. Beta-diversity metrics was calculated using qiime2. Distance metrics calculated included weighted and unweighted UniFrac50 and Bray–Curtis dissimilarity index. Data was visualized using principal coordinate analysis (PCoA) plots and boxplots which were generated using ggplot2^60^ within R version 3.5.2.

A Kruskal–Wallis test with Dunn's post-hoc test was used to identity individual taxa differentially represented across treatment groups and assess variations in alpha diversity between treatment groups. Distance based permutation multivariate analysis of variance (PERMANOVA)^61^ was performed to test the null hypothesis that there were no differences in the microbial community structure across treatment at a significance level of *P* = 0.05 based on 999 permutations. Resulting *P* values were false discovery rate (FDR) corrected 0.05 as the cut off. The corrected *P* values are presented as q-values.

MaAsLin2 is a statistical framework that performs boosted, additive general linear models between clinical data and bacterial abundance to find associations between clinical data (categorical or continuous) and microbial taxa^62^. MaAsLin2 was used to find associations between bacterial taxa and RFI phenotype. Dietary phase, age (days) and breed were included in the statistical model in order to elucidate microbes which were associated with RFI independent of any other these factors. A FDR *P* value of < 0.10 was used as significance cut-of. MaAsLin2 was also performed to show the potential association between SCFA and alpha diversity metrics with RFI.
